# Use of automatic SQL generation interface to enhance transparency and validity of health-data analysis

**DOI:** 10.1016/j.imu.2022.100996

**Published:** 2022-06-25

**Authors:** Kavishwar B. Wagholikar, David Zelle, Layne Ainsworth, Kira Chaney, Alexander J. Blood, Angela Miller, Rupendra Chulyadyo, Michael Oates, William J. Gordon, Samuel J. Aronson, Benjamin M. Scirica, Shawn N. Murphy

**Affiliations:** a Harvard Medical School, Boston, MA, USA; b Brigham and Women’s Hospital, Boston, MA, USA; c Massachusetts General Hospital, Boston, MA, USA; d Mass General Brigham, Boston, MA, USA

**Keywords:** Structured query language, Validity of analysis, Reproducibility of analysis, Databases, Graphical user-interface

## Abstract

Analysis of health data typically requires development of queries using structured query language (SQL) by a data-analyst. As the SQL queries are manually created, they are prone to errors. In addition, accurate implementation of the queries depends on effective communication with clinical experts, that further makes the analysis error prone. As a potential resolution, we explore an alternative approach wherein a graphical interface that automatically generates the SQL queries is used to perform the analysis. The latter allows clinical experts to directly perform complex queries on the data, despite their unfamiliarity with SQL syntax. The interface provides an intuitive understanding of the query logic which makes the analysis transparent and comprehensible to the clinical study-staff, thereby enhancing the transparency and validity of the analysis. This study demonstrates the feasibility of using a user-friendly interface that automatically generate SQL for analysis of health data. It outlines challenges that will be useful for designing user-friendly tools to improve transparency and reproducibility of data analysis.

## 1. Introduction

Reporting on clinical studies typically requires efforts of a data analyst to decipher the study-database’s schema. The data-analyst typically develops Structured Query Language (SQL) queries for finding patient-cohorts that satisfy particular clinical-criteria. However considerable domain knowledge is often required to implement the clinical-criteria SQL, and hence the data-analyst is often paired with a clinical expert who can explain the criteria in terms of the available data elements. This collaboration often requires weeks or months to yield a set of SQL statements that produce the desired analysis. However, these SQL statements are only amenable to modification or troubleshooting by the data-analyst. The clinical expert cannot modify the SQL queries due to unfamiliarity with the SQL, thus rendering it difficult to independently validate the queries and the resulting analysis.

We propose an alternative approach wherein a graphical interface that automatically generates and executes SQL queries is used to perform the analysis. We investigate the feasibility of the proposed approach for analysis of a population-health program.

## 2. Methodology

We implemented two approaches to generate an analytical report for a population-health program at Mass General Brigham (MGB) in Boston [1–4]. This study included health-data for patients receiving care at MGB and was approved by institutional ethics review board.

**Fig. 1.**
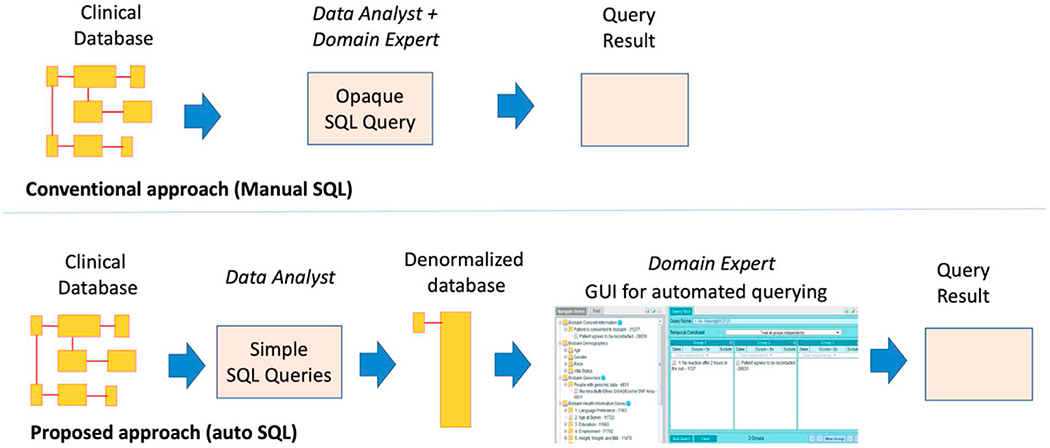
In the conventional approach the data-analyst developed a SQL query to generate the report, while in the proposed auto-SQL approach the data-analyst first denormalized the study database and then the domain expert used the i2b2-webclient graphical-user-interface that automatically generated the SQL for performing the analysis.

As shown in Fig. 1, first the conventional approach was implemented wherein a data-analyst developed a SQL query to generate the report. Next we implemented a second approach, wherein a domain expert used the ‘Informatics for Integrating Biology and the Bedside’ (i2b2) platform’s graphical-user-interface that automatically constructs and executes the SQL queries for computing the report [5]. In the second approach, the data-analyst is required to first denormalized the clinical-database into a star-schema that is compatible with the i2b2 platform. Detailed methodology is described below.

### 2.1. Informatics for Integrating Biology and the Bedside (i2b2) platform

I2b2 platform is deployed at major academic health institutions for querying for electronic health records (EHR) [5,6]. It has been deployed as critical component of research networks [7–10], and is used for a widely for clinical-trial enrollment [11], biobanking [12–14], and epidemiological analysis [15–19]. The platform provides a user-friendly interface that allows researchers with no expertise in Information Technology to find patient cohorts using EHR data.

### 2.2. Study data

The clinical database in this study included data for 7782 patients that were enrolled into the population-health program for lipid-therapy optimization [20]. The study involved use of an algorithm-based electronic decision support tool that leveraged patient data to identify and optimize therapy for patients within primary care practices at MGB. Program staff contacted eligible patients by phone to provide suggestions for optimizing medications. Data-entry was performed using custom software developed for the study, and the data was recorded in a Microsoft SQL database [2].

We performed analysis to quantify the proportion of patients, that were contacted by the program staff before exiting from the program. The patients were segmented into groups based on the reason for exit from the program, as shown in Table 1.

### 2.3. Conventional Manual-SQL approach

The data-analyst closely worked with the clinical experts to identify the database tables and columns required to generate the analytical-report (see Table 2).

The analyst developed a large SQL query that performed the following:

1. Join the necessary tables with the identity keys
2. Filter out errors in the data
3. Group the patients by the exit-reasons
4. Determine if the patients were contacted by program staff before exit from the program
5. Output the result as a table

### 2.4. Auto-SQL approach

In the second approach, a data-analyst from the study team – different from the one who performed the manual approach – communicated with clinical experts to identify the data elements required for the report. Next the analyst developed SQL code to extract the required data in a denormalized form. Denormalization involved merging of all the involved tables to create two tables— one table containing all the patient data, and the second table serving as the meta-data or dictionary of the elements in the patient data. These tables were imported respectively into the observation-fact and concept-dimension tables in the i2b2-platform [21,22].

We installed the i2b2-wildfly and i2b2-webclient docker containers to facilitate querying of the de-normalized data [23]. The i2b2 graphical user interface (GUI) was used to obtain the counts in each cell of the analytical-report.

**Fig. 2.**
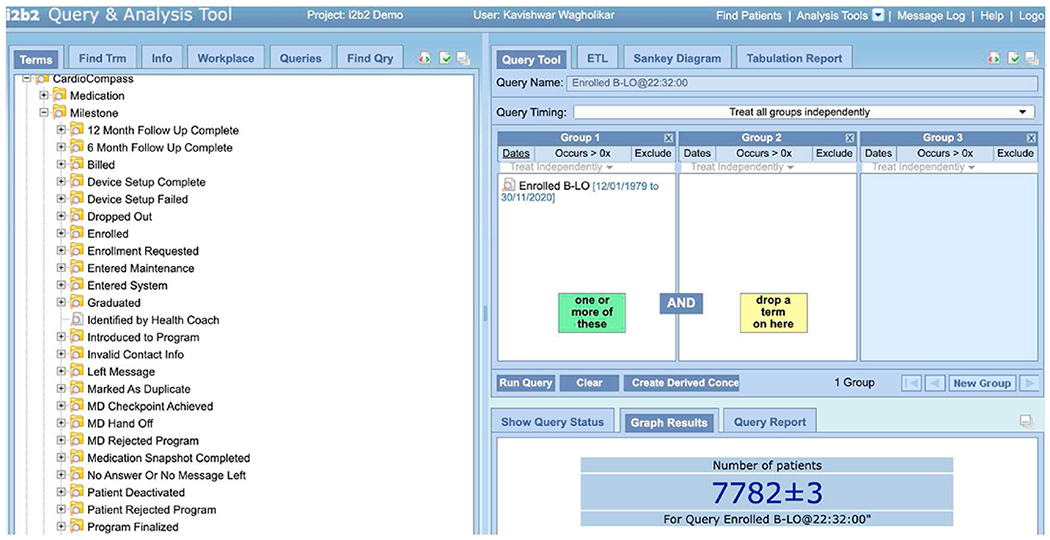
Graphical query interface from the i2b2 platform. The criteria for querying can be easily constructed by dragging terms from the hierarchical tree structure on the left to the widgets on the right. The SQL query is automatically generated in the back-end, which enables clinical staff that are not familiar with SQL to perform complex queries on the data.

Specifically, to compute each of the counts in the report, a clinical expert created queries by dragging the appropriate terms from the i2b2 concept-tree/ontology into the query panes (see Fig. 2). The i2b2 platform automatically generated and executed the SQL code and presented the results in the GUI. The latter were collated by the clinical expert into a table (see Table 2), and compared to the table generated by the conventional approach.

## 3. Results

The auto-SQL approach could be successfully applied to generate the analytical table. Specifically, the query logic required to generate the report could be implemented using the graphical interface for i2b2. The advanced temporal querying feature in the i2b2 GUI was used, as the analysis involved finding if the patients were contacted before or after they exited from the program.

The tables generated by both the approaches differed slightly in the cell counts. We performed an error analysis to investigate the differences in the counts and identified two sources of the errors—test patients and patients from the pilot-stage were imported in the second approach. The test patients could be excluded by applying a concept-filter and the patients from pilot stage could be excluded by excluding patient records before a cut-off date. After applying these corrections, the resulting tables were a near exact match.

## 4. Discussion

We implemented two approaches to generate an analytical report for a clinical study. First, the conventional manual-SQL approach wherein the data-analyst hand-coded a SQL query to generate a report. And second auto-SQL approach wherein a graphical-user-interface that automatically generated SQL queries was used for generating the report.

Our results demonstrate that the report generated by the auto-SQL closely approximated the report generated by manual-SQL approach. This was useful to validate the report. As automatically generated SQL is not prone to syntactical errors that occur in manually developed SQL, successful replication of the result also assured the study staff that there are no errors due to syntactical mistakes or incorrect query criteria in the manual SQL query.

Our study demonstrated that the use of i2b2-GUI was sufficient in implementing all the involved query criteria, although the advanced temporal querying feature was required to implement some of the queries. As the i2b2-GUI can be used without any knowledge of SQL programming the report can be generated by a wider set of staff members that enhances transparency of the analysis, which ultimately ensures validity of the results.

However, a major limitation of i2b2-GUI it can only be used to generate reports that comprise of patient counts. This is because i2b2-platform has been primarily developed for cohort identification. Hence analysis that involve the filtering data for particular clinical-concept, e.g. highest blood-cholesterol or last blood cholesterol cannot be performed in i2b2. Similarly aggregate statistics at level of patient-cohorts, e.g. average blood-cholesterol of male patients, cannot be performed in i2b2. Another limitation is that the i2b2-GUI serves results as a patient count that corresponds to a cell in a tabular report. Hence multiple queries are required to compute all the cells in a tabular report.

There is a wide spectrum of approaches that offer varying degrees for assistance to automatically generate SQL queries, including SQL editors, Tableau, and programming libraries [24]. At one end the Integrated Development Environments (IDEs) for SQL offer generation of SQL snippets to query a given database table. However, the IDEs do not provide automated assistance for generating SQL that joins tables. Use of the SQL IDEs requires some familiarity with SQL. The i2b2-GUI is stands-out in its [25] low requirement of familiarity of SQL, as it leverages the star-schema to automatically generate the SQL.

The i2b2-query tool has advanced SQL generation capabilities as compared to the snippets generated by Integrated Development Environments (IDEs). IDEs typically inspect a database schema to generate sample SQL statements for data selection, insertion, updating and deletion. In contrast i2b2-query tool translates complex query conditions into SQL queries. The i2b2-GUI is designed to query the star schema which provides a generic model of electronic health record data, with all the patient-data stored in the central fact table, and the meta-data and contextual information stored in dimension tables. The wide use of the i2b2-query tool over the last decade is evidence of how the tool is friendly for clinical users with no training in informatics or programming. Although the capability of i2b2 platform to identify patient cohorts with the help of a graphical interface is well known, our study systematically demonstrates the use of i2b2 to perform data-analysis. I2b2 is typically deployed as an enterprise application for researchers to find patients for clinical research, and our study demonstrates how the use of i2b2 can be extended to support the use-case of project specific data analysis.

Another aspect of the auto-SQL generation by the i2b2-query tool is that the SQL is generated and executed at runtime. This contrasts with tooling like Tableau where the SQL query is first provided by the user for creating the report template and then executed at runtime [26]. However the main advantage of Tableau is that it provides interactive visualization by grouping and rendering the results as charts [27,28]. Summarily, i2b2-query tool has advanced dynamic query generation features, with capability of rendering only basic and limited visualization, while Tableau has relatively simple SQL generation capabilities, but advanced functionality for user-interaction and rendering visualizations.

Previous research on the star schema of i2b2 has shown that the i2b2 schema is interoperable with other standard schemas including OMOP, FHIR, and PCORNet [29,30]. The deployment of i2b2 at health institutions involves transformation of the data from institution or vendor specific EHR schemas into the i2b2 star schema. As the local schemas typically have a large number of tables, transforming such data into the two central tables in i2b2 – observation-facts and concept-dimension, essentially is a denormalization process, which increases the redundancy of data, while making it easy to query by reducing the number of joins. Denormalization in the star schema is known to significantly improve the performance of query execution [31,32]. The transformation for de-normalizing the local-schema only needs to be performed once before loading the data into i2b2, and the transformation does not need to be repeated when new data comes in unless there is a modification to the schema. If there is an update to the schema, the transformation needs to be modified to accommodate the changed data elements.

There are several short-comings in the i2b2 platform that need to be addressed before it can be used as an analytical or reporting tool – i2b2 is designed to generate a count given a cohort definition. Reporting requires tabulation of several counts to provide a comparison across cohorts, and it is cumbersome to generate the individual counts using the i2b2 to manually populate the cells in a table. Hence functionality to generate count tables is necessary to support the use of i2b2 for reporting. Moreover, it will be useful if the tables can be visualized using the appropriate chart or bar diagrams, and if the reports can be scheduled to run at period intervals.

A major limitation of our study is that we performed our analysis in the context of generating a single analytical report. It would be helpful to replicate our study design for reproducing multiple other reports. In addition, surveying users with examples of queries performed in real-world settings will also be helpful to objectively establish the usefulness of the i2b2-UI.Conclusion.

Our study demonstrates the feasibility of using user-friendly interfaces that automatically generate-SQL to perform analysis of health data. We discussed limitations in the existing tools that offer automated SQL support to non-technical users. Our report will be useful for designing user-friendly tools to improve transparency and reproducibility of data analysis.

## Figures and Tables

**Table 1:** Reasons for exit from the Lipid therapy optimization program.

Exit category	Description
Dropped out	Patient dropped out of the study before their therapy could be optimized
Entered maintenance	Patient successfully completed the program
MD-handoff	Patient was referred to their primary cardiologist for management
MD rejected program	Patient’s care provider did not consent to including the patient in the optimization program
Unreachable	Patient could not be contacted by the study team.

**Table 2:** Analytical report generated by conventional approach.

Milestones	Not titrated before exit	Titrated before exit	Row total
Dropped out	285	107	392
Entered maintenance	704	876	1580
MD-handoff	59	60	119
MD rejected program	18	5	23
Unreachable	718	278	996
Total	1784	1326	3110

## Data Availability

The datasets used in the current study cannot be made publicly available due to privacy regulations. For any questions related to the datasets please email kwagholikar@mgh.harvard.edu.
